# Preventive effects of *Chlorella* on skeletal muscle atrophy in muscle-specific mitochondrial aldehyde dehydrogenase 2 activity-deficient mice

**DOI:** 10.1186/1472-6882-14-390

**Published:** 2014-10-11

**Authors:** Yuya Nakashima, Ikuroh Ohsawa, Kiyomi Nishimaki, Shoichiro Kumamoto, Isao Maruyama, Yoshihiko Suzuki, Shigeo Ohta

**Affiliations:** Department of Research and Development, Chlorella Industry Co. Ltd, 1343 Hisatomi, Chikugo, Fukuoka 833-0056 Japan; Department of Biochemistry and Cell Biology, Institute of Development and Aging Sciences, Nippon Medical School, 1-396 Kosugi-machi, Nakahara-ku, Kawasaki, Kanagawa 211-8533 Japan; Biological Process of Aging, Tokyo Metropolitan Institute of Gerontology, 35-2 Sakae, Itabashi-ku, Tokyo 173-0015 Japan

**Keywords:** Muscle atrophy, *Chlorella*, Aldehyde dehydrogenase 2, Mitochondrial cytochrome *c* oxidase, Oxidative stress

## Abstract

**Background:**

Oxidative stress is involved in age-related muscle atrophy, such as sarcopenia. Since *Chlorella*, a unicellular green alga, contains various antioxidant substances, we used a mouse model of enhanced oxidative stress to investigate whether *Chlorella* could prevent muscle atrophy.

**Methods:**

Aldehyde dehydrogenase 2 (ALDH2) is an anti-oxidative enzyme that detoxifies reactive aldehydes derived from lipid peroxides such as 4-hydroxy-2-nonenal (4-HNE). We therefore used transgenic mice expressing a dominant-negative form of ALDH2 (ALDH2*2 Tg mice) to selectively decrease ALDH2 activity in the muscles. To evaluate the effect of *Chlorella,* the mice were fed a *Chlorella*-supplemented diet (CSD) for 6 months.

**Results:**

ALDH2*2 Tg mice exhibited small body size, muscle atrophy, decreased fat content, osteopenia, and kyphosis, accompanied by increased muscular 4-HNE levels. The CSD helped in recovery of body weight, enhanced oxidative stress, and increased levels of a muscle impairment marker, creatine phosphokinase (CPK) induced by ALDH2*2. Furthermore, histological and histochemical analyses revealed that the consumption of the CSD improved skeletal muscle atrophy and the activity of the mitochondrial cytochrome c oxidase.

**Conclusions:**

This study suggests that long-term consumption of *Chlorella* has the potential to prevent age-related muscle atrophy.

## Background

Excessive production of reactive oxygen species (ROS) causes oxidative damage to DNA, proteins, and lipids. This damage accumulates with age in various organs, including the skeletal muscle, as observed in both humans [1–4] and animal models [5, 6]. Thus, it is hypothesized that some antioxidants may aid in preventing age-related disorders, including muscle atrophy, such as sarcopenia.

We have sought to develop animal models with enhanced oxidative stress and various impairments that are affected by age [7, 8]. Aldehyde dehydrogenases (ALDH) catalyze the conversion of reactive aldehydes to carboxylates [9]. Mitochondrial ALDH2 is known to oxidize acetaldehyde produced from ethanol into acetate [10], and a single nucleotide polymorphism in this gene found in Asian populations, ALDH2*2, produces a dominant-negative protein that prevents this activity. We have previously revealed, through a molecular epidemiological analysis, that a higher concentration of lipid peroxides are present in the sera of ALDH2-deficient females than in those expressing active ALDH2 [11]. Furthermore, we demonstrated that ALDH2 deficiency is a risk factor for late-onset Alzheimer’s disease [12], suggesting a role for this polymorphism in human diseases. Recently, ALDHs have emerged as an important enzyme in a variety of human pathologies. ALDH2 dysfunction contributes to a variety of human diseases including diabetes, cancer, cardiovascular diseases [13–15], neurodegenerative diseases, stroke, Fanconi anemia, pain, osteoporosis, and the aging process [16].

In previous examinations of ALDH2*2, we showed that mitochondrial ALDH activity was repressed when murine ALDH2*2 was stably expressed in the neuronal cell line PC12. Cells expressing ALDH2*2 were also vulnerable to 4-hydroxy-2-nonenal (4-HNE), and treatment with 4-HNE or antimycin A was shown to induce cell death [17, 18]. Additionally, ALDH deficiency enhanced oxidative stress through a vicious cycle [8].

A Tg mouse model expressing ALDH2*2 specifically in the brain decreased the ability of mice to detoxify 4-HNE in cortical neurons and accelerated the accumulation of 4-HNE in the brain [7]. Consequently, these mice presented with age-related neurodegeneration accompanied by memory loss after maturation. Mice deficient in muscle-specific mitochondrial ALDH2 activity were also developed by inducing the transgenic expression of ALDH2*2 under the control of the actin promoter [8]. These model animals will be helpful in investigating the antioxidant properties of health foods *in vivo*, as well as in studies examining the prevention of oxidative stress-related muscle atrophy.

Sarcopenia is the decline of muscle mass and strength that occurs with aging [19]. Since the progression of sarcopenia induces significant physical depression [20–22], this condition increases the risk of fractures due to fall and the possibility of becoming bedridden in elderly people. A central mechanism in the pathogenesis of sarcopenia is oxidative stress [23], which has been detected by the accumulation of several oxidative stress markers. These aldehyde species, which primarily include malondialdehyde (MDA) and 4-HNE, are spontaneously generated from lipid peroxides [24]. Interestingly, 4-HNE is a strong electrophile that rapidly reacts with most biomolecules [25].

*Chlorella*, a unicellular green alga, contains a variety of nutritional components that are rich in protein, fatty acids, dietary fiber, chlorophylls, minerals, vitamins, and carotenoids. Thus, dried *Chlorella* powder or extracts in hot water have long been used as a health supplement in Asia. It has been reported that *Chlorella* elicits various immunopharmacological effects [26–28] and functions as an antioxidant *in vitro* and *in vivo* [29–37]. This is likely because it is rich in carotenoids and other antioxidants, including lutein, β-carotene, α-tocopherol, and ascorbic acid. We have already demonstrated that long-term consumption of *Chlorella* did not significantly affect the weight of any organs in wild type rats [38], and that long-term *Chlorella* consumption prevents oxidative stress, age-dependent cognitive decline, and central nervous system disorders in Tg mice expressing ALDH2*2 in the brain [39]. Thus, *Chlorella* supplements have displayed antioxidant effects in a variety of animal experiments. However, it is unknown whether the consumption of *Chlorella* has an antioxidant effect in muscle tissues. Importantly, inflammation may cause sarcopenia in addition to oxidative stress. Since *Chlorella* extract slight decreased the expression of the pro-inflammatory cytokine IL-6 in mice [40], we here focused on the effects of oxidative stress.

In this study, we fed *Chlorella* to mice expressing a dominant negative, muscle-specific form of mitochondrial ALDH2 for 6 months. Our findings suggest that this supplement has the potential to mitigating skeletal muscle atrophy.

## Methods

### Animals and treatment

Tg mice expressing ALDH2*2 in the skeletal muscle were previously reported [8]. Heterozygous male mice were mated with female C57BL/6 mice (Kyudo, Fukuoka, Japan). Genotyping of the resulting mice was performed by PCR using genomic DNA isolated from the tail tip and 2 sets of combinatorial primers (5′-CGTGCTGGTTATTGTGCTGTCTCA-3′ and 5′-GAAGGGTTGACGGTGGGAAATGTT-3′; 5′-TGGCGTGGTCAATATCGTTCCC-3′ and 5′-GAGCTTGGGACAGGTAATTGGC-3′) in order to amplify the exogenous ALDH2*2 gene. Sixteen heterozygous ALDH2*2 Tg male mice were identified and used in the experiments described in this report. These mice were divided into 2 groups of 8 mice at 8 weeks of age. By this time, the two groups had developed a uniform average of body weight. One group was fed a basic diet (Control group), while the other group was fed a 1% *Chlorella*-supplemented diet (CSD group) for 6 months. The experimental dietary compositions were based on the AIN-93 M [41], and are shown in Table 1. Wild type littermates were also fed the basic diet. All animals were kept on a 12 h light/dark cycle with *ad libitum* access to water and food throughout the experimental period, and body weight and food intake were measured at the end of each month. This study was approved by the Animal Care and Use Committee of Chlorella Industry Co., Ltd., according to the National Institutes of Health published guidelines.

**Table 1:** **Composition of experimental diets supplemented with** ***Chlorella*** **powder**

Ingredients	Control and wild type groups	CSD group
Casein	140.0	138.6
L-Cysteine	1.8	1.8
Corn starch	465.7	460.5
Dextrinized corn starch	155.0	153.5
Sucrose	100.0	99.0
Soybean oil	40.0	39.6
Fiber	50.0	49.5
Vitamin mix (AIN-93-VM)^a^	10.0	10.0
Mineral mix (AIN-93-MX)^a^	35.0	35.0
Choline bitartrate	2.5	2.5
Tert-butylhydroquinone	0.008	0.008
*Chlorella* powder	0	10.0
Total weight (g)	1000	1000
Energy (kJ)	16362	16358

Quantity expressed as g/kg diet.

Control ALDH2*2 mice were fed a basal diet, CSD ALDH2*2 mice a *Chlorella*-supplemented diet, and Wild type C57BL6 mice basal diet.

^a^Vitamins and minerals based on AIN-93 M formulation [41].

### *Chlorella*powder

The *Chlorella* powder used to prepare the CSD was from the *Parachlorella beijerinckii* CK-5 strain, and was cultured, dried, and powdered by Chlorella Industry Co., Ltd. The general characteristics and composition of the *Chlorella* powder is shown in Table 2.

**Table 2:** ***Chlorella*** **powder composition**

Component	***Chlorella***powder (per 100 g powder)
Protein	65 g
Carbohydrate	0.9 g
Fat	11.9 g
Dietary Fiber	10.2 g
Ash	6.6 g
Moisture	5.4 g
Lutein	274 mg
α-Carotene	12 mg
β-Carotene	118 mg
Ascorbic acid	46 mg
α-Tocopherol	32.8 mg

### Sample collection

All mice were anesthetized and sacrificed prior to blood sample collection from the hepatic portal vein. Plasma was subsequently obtained by centrifugation at 3000 × *g* for 15 min at 4°C. The liver, kidneys, heart, lung, spleen, right gastrocnemius muscle, and adipose tissue (epididymal) of each mouse were quickly excised and weighed. For histological and histochemical studies, the left gastrocnemius muscle was frozen in hexane with Optimal Cutting Temperature (OCT) Compound (Sakura Finetek Japan, Tokyo, Japan) at -80°C. The quadricep muscle was washed with cold phosphate buffered saline (pH 7.4) and frozen in liquid nitrogen. All samples were stored at -80°C until use.

### Plasma analysis

The plasma activities of creatine phosphokinase (CPK) and creatine phosphokinase-MB (CKMB) were measured using a DRI-CHEM autoanalyzer (FUJIFILM, Tokyo, Japan).

### Bone density measurement

For computed tomography analysis of bone density, the whole bodies of the mice were scanned using a LaTheta LCT-100 experimental animal computed tomography system (Aloka, Tokyo, Japan). Contiguous 1-mm thick slices were used for quantitative assessment using LaTheta software (ver 1.00). Bone density was evaluated quantitatively.

### Analysis of urinary oxidative stress

In order to measure urinary oxidative stress markers, mice were placed in a urine-sampling cage for 12 h at 2 and 4 months after the experiment began, and urine was collected. Urinary isoprostane levels were determined using a urinary isoprostane F_2t_ ELISA kit (JaICA, Fukuroi, Japan), according to the manufacturer’s instructions. The creatinine concentration of each sample was measured using a LabAssay Creatinine kit (Wako, Osaka, Japan). Urinary isoprostane levels were normalized against creatinine concentration.

### Measurement of oxidative stress in skeletal muscle

MDA and 4-hydroxyalkenals (HAE) levels in the quadriceps muscles were determined using a Bioxytech LPO-586 assay kit (OxisResearch, Oregon, USA). Briefly, pieces of the quadriceps muscle were homogenized in phosphate-buffered saline (pH 7.4) containing 5 mM butylated hydroxytoluene. After homogenization, the samples were centrifuged at 3000 × *g* for 10 min at 4°C, and the clear supernatants were subjected to the LPO-586 assay. MDA and HAE levels were assayed using the methanesulfonic acid solvent procedure according to the manufacturer’s instructions. The LPO-586 assay is based on the reaction of a chromogenic reagent, *N*-methyl-2-phenylindole, with MDA and HAE at 45°C. These compounds react with *N*-methyl-2-phenylindole to yield a stable chromophore with a maximal absorbance at 586 nm. The absorbance of the resultant samples was measured at 586 nm. The protein concentration of each sample was measured using a Pierce BCA protein assay kit (Thermo Scientific, Rockford, USA). MDA and HAE levels were normalized against protein concentration.

### Histological and histochemical studies

Frozen gastrocnemius muscle samples were sliced into sections (8-μm thick) and mounted on silane-coated glass slides. Frozen sections were dried and stained with hematoxylin and eosin (H&E). For enzymatic cytochrome *c* oxidase histochemical staining, frozen sections were dried and incubated in 0.1 mol/L sodium phosphate (pH 7.4), 0.5 mg/ml 3,3-diaminobenzidin (DAB, Wako), 130 μg/ml catalase (Nacalai tesque, Kyoto, Japan), and 1 mg/ml cytochrome *c* (Nacalai tesque) at room temperature for 60 min. The cross-sectional area of the gastrocnemius muscle cells and the area of stained cytochrome *c* oxidase were calculated using the Image J (ver1.41; National Institutes of Health, Bethesda, MD), and they are presented as the percent ratio (%) versus wild type from 3 different points for each mouse.

### Statistical analysis

All values shown are the mean ± SD. One-way ANOVA (Fisher’s PLSD test) followed by contrast testing was used to compare the data from multiple groups. Relationships between given variables were examined by linear regression analysis and the Pearson correlation coefficient. All experiments were examined in a blinded fashion, and statistical significance was accepted as *p* <0.05.

## Results

### Consumption of a *Chlorella*-supplemented diet reduces oxidative stress and reverses skeletal muscle impairment

**Figure 1:**
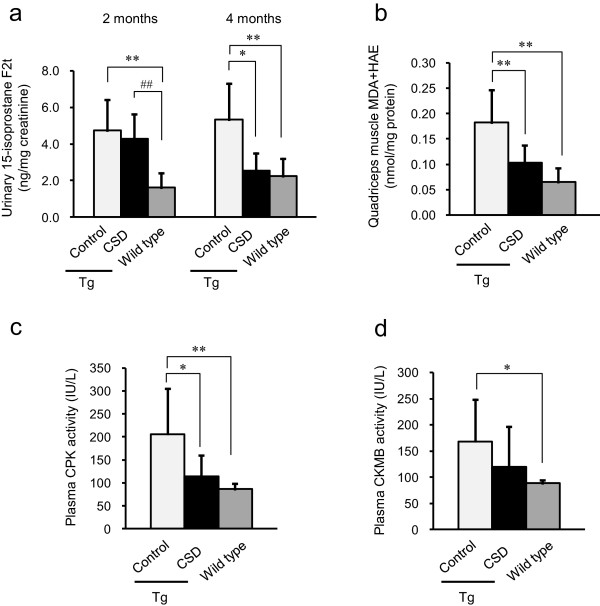
**Consumption of** ***Chlorella*** reduced oxidative stress and muscle tissue injury in ALDH2*2 Tg mice. Wild type (*n* =7) and control ALDH2*2 Tg mice (*n* =8) were fed a basal diet, while the CSD ALDH2*2 Tg mice received a *Chlorella*-supplemented diet (*n* =8). Urinary 15-isoprostane levels were measured 2 and 4 months after the initial *Chlorella* administration **(a)**. After 6 months of consuming the *Chlorella* diet, MDA and HAE were measured in the quadriceps muscle **(b)**. The levels of CPK **(c)** and CKMB **(d)** detectable in plasma were measured 6 months after initial *Chlorella* administration. Values are the mean ± SD, *p <0.05 and **p <0.01: significant vs. Control; ^#^ *p <*0.01: significant vs. Wild type.

To investigate oxidative stress in the ALDH2*2 Tg mice, we examined the levels of the urinary oxidative stress marker isoprostane, 2 and 4 months after initiating CSD feeding. Although no difference was observed between the CSD and Control groups after 2 months, the level of urinary isoprostane was decreased in mice fed the CSD for 4 months (Figure 1a). In addition, we measured a second oxidative stress marker, MDA and HAE, in quadriceps muscle after 6 months of *Chlorella* consumption, and found that the accumulation of the oxidative stress marker was notably suppressed in these mice (Figure 1b).

Excess oxidative stress is known to impair muscle. To evaluate the extent of muscle impairment in the ALDH2*2 Tg mice, we measured the levels of CPK and CKMB expression in plasma. The ALDH2*2 Tg mice had increased levels of both proteins; however, CPK levels were significantly reduced after CSD administration (Figure 1c). A trend toward reduced of CKMB levels was also observed (Figure 1d). These results suggest that the consumption of *Chlorella* reduces oxidative stress and reverses skeletal muscle impairment in ALDH2*2 mice (Figure 1).

### Effect of *Chlorella*-supplemented diet consumption on body size and skeletal muscle atrophy

**Figure 2:**
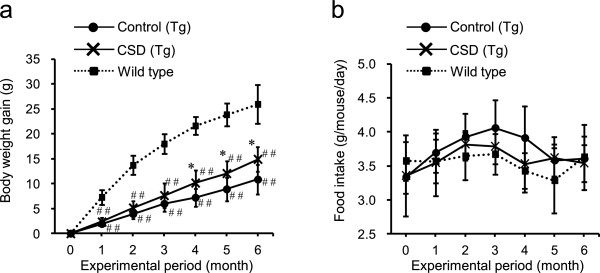
**Effects of** ***Chlorella*** **consumption on body weight gain and food intake.** All groups were fed the respective diets shown in Table 1, beginning at 2 months of age. Wild type (*n* =7) and control ALDH2*2 Tg mice were fed a basal diet (*n* =8), while the CSD ALDH2*2 Tg mice received a *Chlorella*-supplemented diet (*n* =8). Body weights **(a)** and food intake **(b)** were measured at the end of each month. Values are the mean ± SD, *p <0.05: significant vs. Control; ^##^ *p <*0.01: significant vs. Wild type.

One month following the initiation of the experimental diets, wild type mice were observed to have a significantly increased body weight compared to ALDH2*2 Tg mice (Figure 2a), suggesting that muscle-specific ALDH2 deficiency has a negative impact on body size. Compared to the consumption of a basic diet, CSD consumption for 4 months resulted in a greater body size in the ALDH2*2 Tg mice (Figure 2a). Importantly, no differences in food intake among any of the groups were observed (Figure 2b), indicating that the body weight of the mice in the CSD group was restored in a food intake-independent manner.

The weight of the organs and bone density of the experimental mice were also measured after 6 months of consuming the respective diets. The weights of both the gastrocnemius muscles and epididymal fat of the CSD group were significantly heavier than those of the Control group (Table 3). In addition, the CSD appeared to suppress the decline in bone density observed in the Control group (Table 3). These results suggest that the consumption of *Chlorella* is beneficial for the growth of ALDH2*2 Tg mice.

**Table 3:** **Effects of** ***Chlorella*** **consumption on bone density and organ weights**

	ALDH2*2 Tg		
	Control group	CSD group	Wild type group
Bone density (mg/cm^3^)	410.9 ± 24.0^#^	426.5 ± 21.8	442.0 ± 21.5
Organ weights (mg)			
Liver	1230.5 ± 134.6^##^	1265.2 ± 140.0^##^	2082.4 ± 391.9
Kidney	224.4 ± 37.6	238.4 ± 25.5	237.9 ± 32.6
Heart	129.3 ± 16.0^##^	135.1 ± 7.6^##^	165.5 ± 6.8
Lung	212.8 ± 23.3^##^	212.2 ± 27.0^##^	263.8 ± 50.9
Spleen	54.0 ± 6.9^##^	60.5 ± 9.4^##^	80.1 ± 11.1
Gastrocnemius muscle	107.9 ± 10.8^##^	129.8 ± 19.6*^,##^	189.0 ± 8.4
Epididymal fat	521.7 ± 265.3^##^	824.2 ± 144.9*^,##^	1044.8 ± 174.4

Control ALDH2*2 mice were fed a basal diet (*n* =8), CSD ALDH2*2 mice a *Chlorella*-supplemented diet (*n* =8), and Wild type C57BL6 mice a basal diet (*n* =7). Bone density and organs weight was measured at 8 months of age, after 6 months of experimental diet consumption. Values are mean ± SD, *p <0.05: Significant vs. Control; ^#^ *p* <0.05*,* ^##^ *p* <0.01: Significant vs. wild type.

**Figure 3:**
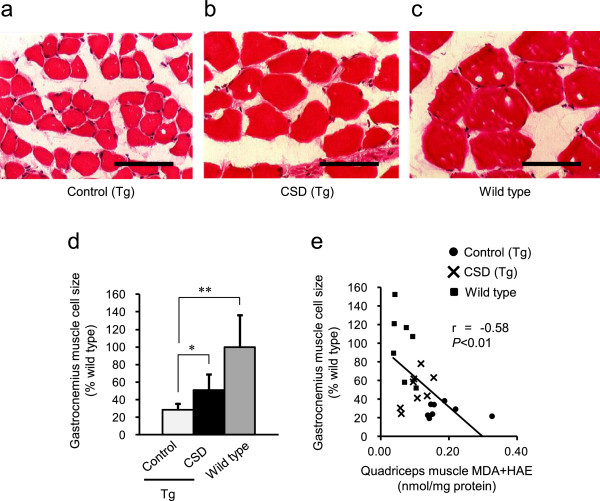
***Chlorella*** consumption prevents skeletal muscle atrophy in ALDH2*2 Tg mice. The cross-sectional areas of gastrocnemius muscle cells were stained with H&E 6 months after the initial administration of *Chlorella* in the diet. The scale bar indicates 100 μm **(a–c)**. Gastrocnemius muscle cell size was measured, and is presented as the percent ratio (%) versus Wild type **(d)**. Gastrocnemius muscle cell size and MDA and HAE had a significant negative correlation **(e)**. Values are the mean ± SD, *p <0.05 and **p <0.01: significant vs. Control.

To examine the effects of CSD consumption on muscle atrophy, we measured the cross-sectional area of the gastrocnemius muscle cells in our experimental mice using H&E staining. The ALDH2*2 Tg mice exhibited a significantly higher cross-sectional area of gastrocnemius muscle cells after consuming the CSD for 6 months than the control mice (Figure 3a–d), suggesting that consumption of *Chlorella* improved skeletal muscle atrophy. Furthermore, when the correlation between oxidative stress in quadriceps and gastrocnemius muscle atrophy was examined, we found that the relative cross-sectional areas of the gastrocnemius muscle cells were negatively correlated with the expression level of MDA and HAE (r = -0.58, *p* <0.01, Figure 3e).

### Protective effects of *Chlorella*-supplemented diet consumption on mitochondrial dysfunction in ALDH2*2 Tg mice

**Figure 4:**
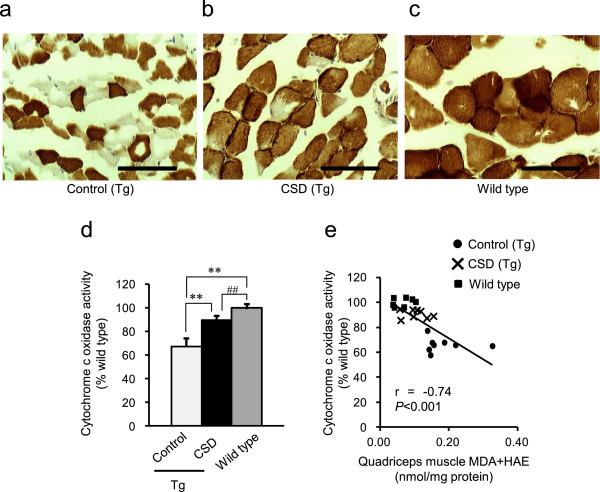
**Effects of** ***Chlorella*** **consumption on mitochondrial cytochrome** ***c*** oxidase activity in ALDH2*2 Tg mice. Enzymatic histochemical staining for cytochrome *c* oxidase in the mitochondria of the gastrocnemius muscle cells 6 months after the initial administration of *Chlorella* in the diet. The scale bar indicates 100 μm **(a–c)**. Gastrocnemius muscle cytochrome *c* oxidase activity was measured and is presented as the percent ratio (%) versus Wild type **(d)**. A significant negative correlation was observed between gastrocnemius muscle cytochrome *c* oxidase activity and MDA and HAE levels in quadriceps muscle **(e)**. Values are the mean ± SD*,* **p <0.01: significant vs. Control; ^##^ *p <*0.01: significant vs. Wild type.

To investigate mitochondrial function in skeletal muscle, we measured cytochrome *c* oxidase activity in the gastrocnemius muscle. When enzymatic histochemical staining for cytochrome *c* oxidase was performed, it was found that notable cytochrome *c* oxidase activity decline was detectable in the gastrocnemius muscle of the ALDH2*2 Tg Control group, while it was significantly decreased in the CSD group (Figure 4a–d). This suggests that *Chlorella* consumption prevented mitochondrial dysfunction in gastrocnemius muscle. In addition, the relative level of cytochrome *c* oxidase activity was negatively correlated with the levels of MDA and HAE (r = -0.74, *p* <0.001, Figure 4e).

## Discussion

The present study demonstrated that long-term intake of *Chlorella* reduced oxidative stress, as determined by changes in oxidative stress markers, including urinary 15-isoprostane and muscle MDA and HAE in transgenic mice with enhanced oxidative stress. This improvement resulted in decreased disintegration of muscle, plasma CPK, and plasma CKMB, and increased bone density, organ weight, muscle cell size, and total body weight. Moreover, mitochondrial activity was improved by long-term intake of *Chlorella* in the oxidative stress-enhanced mice.

Oxidative stress in skeletal muscle is associated with the atrophy, loss of muscle function, and fibers in sarcopenia [23, 42]. Thus, it is important to reduce oxidative stress in our daily lives. Indeed, epidemiological studies of community-dwelling older adults have demonstrated that the low carotenoid level in blood is associated with low skeletal muscle strength and the development of walking disabilities [43]. These reports further indicate that dietary carotenoid intake is efficacious in the prevention age-related muscle disorders.

It is unknown why *Chlorella* was effective in reducing oxidative stress. As *Chlorella* contains various dietary antioxidant substances, including carotenoids and vitamins, it could be a potential dietary source of these compounds. Additionally, *Chlorella* also has a large chloroplast, in which plastoquinone substitutes for ubiquinone as an electron carrier in the photosynthetic electron-transport chain. Plastoquinone has been shown to have greater antioxidant properties than ubiquinone [44] and does not pose a danger for pro-oxidant effects within a range of concentrations [45]. Therefore, the effect of *Chlorella* is likely synergistic between the plastoquinone and carotenoids provided in the CSD, thereby protecting against the impairments observed in ALDH2*2 Tg mice. Indeed, we have previously shown that *Chlorella* consumption reduces oxidative stress (4-HNE) in the dentate gyrus of the hippocampus [39]. In the present study, we have further demonstrated that the consumption of a CSD markedly suppresses oxidative stress in the quadriceps muscle, and that there is a negative correlation between oxidative stress in quadriceps and gastrocnemius muscle atrophy.

The age-dependent accumulation of mitochondrial DNA (mtDNA) mutations, which lead to mitochondrial dysfunction, may be an important contributor to sarcopenia [46, 47]. A causal role for these age-related mtDNA deletion mutations and mitochondrial dysfunction in sarcopenia has been supported by findings that these alterations induce the loss of cytochrome *c* oxidase activity in aged rats, primates, and human skeletal muscle cross sections [48–52]. Conversely, since the stimulation of HNE degradation restored the decline in cytochrome *c* oxidase activity [53], HNE should inhibit cytochrome *c* oxidase activity through the formation of HNE adducts with cytochrome *c* oxidase subunits. These findings indicate that the protection of mitochondrial function, especially with regard to the cytochrome *c* oxidase activity, could be important to prevent sarcopenia. As ALDH2 activity is suppressed in ALDH2*2 Tg mice, they cannot efficiently degrade HNE in muscles [8]. In this study, the consumption of a CSD maintained cytochrome *c* oxidase activity in the gastrocnemius muscle of ALDH2*2 Tg mice, and a negative correlation between cytochrome *c* oxidase activity and MDA and HAE was identified. Therefore, the consumption of *Chlorella* may act to prevent the accumulation of HNE, thereby preventing mitochondrial dysfunction through the protection of cytochrome *c* oxidase activity.

As a lack of protein intake and decreased amino acid muscle protein synthesis leads to a decline in muscle mass, the intake of dietary protein has been recommended to slow and prevent the progression of sarcopenia [54, 55]. With a protein content of approximately 65%, the continuous intake of *Chlorella* may therefore be useful in both enhancing muscle protein synthesis and preventing muscle atrophy. In particular, *Chlorella* contains essential amino acids such as the BCAA valine, leucine, and isoleucine, which are important components of actin and myosin-composing muscle, and may be beneficial in the prevention and treatment of sarcopenia [56, 57].

Since *Chlorella* supplement contains various useful substances, it may not be easy to identify a single substance that exhibits a beneficial effect against muscle atrophy. Supplements with multiple compounds, such as *Chlorella,* may be particularly beneficial because of their synergic effects.

Finally, the present study showed the usefulness of this ALDH2 deficient mouse for evaluating anti-oxidative supplements.

## Conclusions

This study demonstrates that long-term consumption of *Chlorella* in the diet has beneficial effects on body weight, and prevents oxidative stress, muscle atrophy, and mitochondrial dysfunction in ALDH2*2 Tg mice. This suggests that *Chlorella* intake may be useful in the treatment of sarcopenia.

## Abbreviations

ALDH2: Aldehyde dehydrogenase 2
4-HNE: 4-hydroxy-2-nonenal
Tg: Transgenic
ALDH2*2: Dominant-negative form of ALDH2
ALDH2*2 Tg mice: Muscle-specific mitochondrial ALDH2 activity deficient mice
CSD: *Chlorella*-supplemented diet
CPK: Creatine phosphokinase
ROS: Reactive oxygen species
MDA: Malondialdehyde
HAE: 4-hydroxyalkenals
HE: Hematoxylin and eosin
mtDNA: Mitochondrial DNA.
